# Transjugular intrahepatic portosystemic shunts (TIPS) for the prevention of variceal re-bleeding – A two decades experience

**DOI:** 10.1371/journal.pone.0189414

**Published:** 2018-01-09

**Authors:** Theresa Bucsics, Maria Schoder, Magdalena Diermayr, Maria Feldner-Busztin, Nicolas Goeschl, David Bauer, Philipp Schwabl, Mattias Mandorfer, Bernhard Angermayr, Manfred Cejna, Arnulf Ferlitsch, Wolfgang Sieghart, Michael Trauner, Markus Peck-Radosavljevic, Josef Karner, Franz Karnel, Thomas Reiberger

**Affiliations:** 1 Division of Gastroenterology & Hepatology, Department of Internal Medicine III, Medical University of Vienna, Vienna, Austria; 2 Vienna Hepatic Hemodynamic Laboratory, Medical University of Vienna, Vienna, Austria; 3 Department of Biomedical Imaging and Image-guided Therapy, Medical University of Vienna, Vienna, Austria; 4 Department of Radiology, Landeskrankenhaus, Feldkirch, Austria; 5 Department of Surgery, Kaiser-Franz Josef Spital, Vienna, Austria; 6 Department of Radiology, Kaiser-Franz Josef Spital, Vienna, Austria; Medizinische Fakultat der RWTH Aachen, GERMANY

## Abstract

**Background and aims:**

Transjugular intrahepatic portosystemic shunts (TIPS) are used in patients with cirrhosis for the prevention of variceal rebleeding.

**Methods:**

We retrospectively evaluated re-bleeding rate, patency, mortality, and transplant-free survival (TFS) in cirrhotic patients receiving TIPS implantation for variceal bleeding between 1994–2014.

**Results:**

286 patients received TIPS (n = 119 bare metal stents, n = 167 polytetrafluorethylene (PTFE)-covered stents) for prevention of variceal re-bleeding. Mean age was 55.1 years, median MELD was 11.8, and the main etiology of cirrhosis was alcoholic liver disease (70%). Median follow-up was 821 days. 67 patients (23%) experienced at least one re-bleeding event. Patients with PTFE-TIPS were at significantly lower risk for variceal re-bleeding than patients with bare metal stents (14% vs. 37%, OR:0.259; p<0.001) and had less need for stent revision (21% vs. 37%; p = 0.024). Patients with PTFE stent grafts showed lower mortality than patients with bare stents after 1 year (19% vs. 31%, p = 0.020) and 2 years (29% vs. 40%; p = 0.041) after TIPS implantation. Occurrence of hepatic encephalopathy after TIPS was similar between groups (20% vs. 24%, p = 0.449).

**Conclusions:**

PTFE-TIPS were more effective at preventing variceal re-bleeding than bare metal stents due to better patency. Since this tended to translate in improved survival, only covered stents should be implemented for bleeding prophylaxis when TIPS is indicated.

## Introduction

Liver cirrhosis may lead to the development of severe complications including variceal bleeding from esophageal or gastric varices, hepatic encephalopathy (HE), ascites, spontaneous bacterial peritonitis (SBP) and hepatorenal syndrome[1]. These complications occur secondary to portal hypertension and represent the main causes of hospital admission, liver transplantation and death in patients with cirrhosis.[2] The hepatovenous pressure gradient (HVPG), an indirect measure of portal hypertension, has been shown to be the most accurate predictor for variceal bleeding with bleeding events occurs almost exclusively occurring if HVPG exceeds 12mmHg.[3] Conversely, the risk of bleeding can be lowered when HVPG is decreased, e.g. by administering non-selective beta blockers (NSBBs).[4–8] However, in some patients, medical or endoscopic prophylaxis of variceal bleeding is not effective to prevent variceal bleeding or re-bleeding.

Recurrence of gastro-esophageal bleeding within 6 weeks is considered to be a strong predictor of death and thus, death within this time-frame is referred to as ‘bleeding-related death’.[9,10]

The implantation of a transjugular intrahepatic portosystemic shunt (TIPS) effectively reduces intravascular pressure in the portal venous system.[11] According to current guidelines [5,6,8], TIPS is recommend in high-risk patients with acute variceal bleeding after initial medical and endoscopic bleeding control and after failure of secondary prophylaxis with medical and endoscopic therapy.[12] In 1988, the first TIPS-procedure was performed using a metal stent.[13] However, one-year patency of metal stents was reportedly only 55% and thus, there was considerable need for subsequent stent revisions.[14,15] An important milestone was the introduction of (self-) expanded polytetraflouroethylene (ePTFE) stents with significantly improved patency rates.[16,17] However, recent reports on the effectiveness of TIPS to control variceal re-bleeding are limited, especially in unselected patients outside of clinical studies.

Thus, the aim of our study was to report our two decade experience on the effectiveness of TIPS implantations to control variceal re-bleeding in a large real-life cohort at two specialized centers in Vienna.

## Methods

### Patients and study design

Adult patients (age ≥18 years) who underwent a TIPS procedure at two tertiary care centers between 1994–2014 were analyzed for this retrospective cohort study. Patients were included if (i) they had an established diagnosis of liver cirrhosis and portal hypertension, (ii) TIPS insertion was successful, and (iii) they received TIPS as treatment for acute variceal bleeding and/or as prophylaxis for variceal re-bleeding. Patients with previous orthotopic liver transplantation (OLT) or non-cirrhotic portal hypertension as well as patients without baseline laboratory (performed within 1–3 days prior to TIPS implantation) and/or insufficient documentation in medical records were excluded. For this retrospective cohort study were searched fmedical histories for laboratory parameters, reports on the TIPS procedure and any re-interventions thereafter.

Primary end point was re-bleeding rate after TIPS implantation. Need for TIPS revision, bleeding-related mortality and transplant-free survival (TFS) were considered as secondary end-points. This study was approved by the Ethics Committee of the Medical University of Vienna (ECS 1760/2014) and the Ethics Committee of the city of Vienna (MA 15, EK 14–264 VK).

### Parameters

Type of stent (bare metal or ePTFE), age, gender, hospitalization, follow-up time, etiology of liver disease, and bleeding events after TIPS-implantation were recorded. Severity of liver disease was assessed using the Model of End Stage Liver Disease score (MELD).[18,19] Varices were classified as esophageal varices (EV), gastroesophageal varices (GEV) or isolated gastric varices (IGV) according to the last endoscopy reports prior to TIPS implantation. Laboratory parameters were recorded 3–7 days before and after TIPS implantation as well as at the last date of follow-up (FU). We also documented the prescription of non-selective ß-blockers (NSBBs) before and after TIPS implantation. “Rescue TIPS” was defined as stent implantation directly associated with the bleeding event (within 3 days) while “elective TIPS” implantation was defined as defined as stent implantation >3 days after the bleeding.

### Assessment of outcomes and need of TIPS revisions

The development of variceal bleeding, ascites, overt HE, hepatocellular carcinoma (HCC), as well as thromboembolic events and/or portal vein thrombosis (PVT) after TIPS implantation were documented. TIPS revisions (i.e. measures to improve shunt patency, e.g. angioplasty) were registered. In case of OLT after TIPS implantation, date of transplantation was recorded. In case of death, cause and date of death were recorded.

### Statistics

Descriptive statistics were used to report characteristics of patients. Distribution of variables was assessed using the Kolgomorov-Smirnov test. Normally distributed values were shown as mean value ± standard deviation (SD), otherwise as median and interquartile range. To calculate differences between patient groups, t-test (parametric distribution), Mann-Whitney-U test (non-parametric distribution), X^2^ test or Fisher’s exact test were used wherever appropriate. Transplant-free survival (TFS) or the development of complications (i.e. re-bleeding or need for TIPS interventions) were analyzed using the Kaplan-Meier method. Patients were censored at their last follow-up or at OLT. Group comparisons regarding time-dependent outcomes and mortality were calculated using log-rank test. Statistical analyses were performed using IBM SPSS Statistics for Windows, (Version 24.0. IBM Corp, Armonk, NY, USA) and Kaplan-Meier analyses were performed using GraphPad Prism version 7.00 for Windows (GraphPad Software, La Jolla, California, USA).

Competing risk analysis with variceal re-bleeding as event of interest, and liver and death as competing events was performed using the Fine and Gray competing risk regression model (Subdistribution Analysis of Competing Risks; https://CRAN.R-project.org/package=cmprsk) and R version 3.4.1 (R Core Team, R Foundation for Statistical Computing, Vienna, Austria) [20,21] P-values <0.05 were considered statistically significant.

## Results

### Patient characteristics (Table 1, Fig 1)

Overall, 363 patients received TIPS to treat and/or prevent variceal bleeding in secondary prophylaxis. 77 patients had to be excluded due to insufficient records (n = 75), underage (n = 1) or non-cirrhotic etiology (n = 1). Finally, n = 286 patients were enrolled.

**Fig 1:**
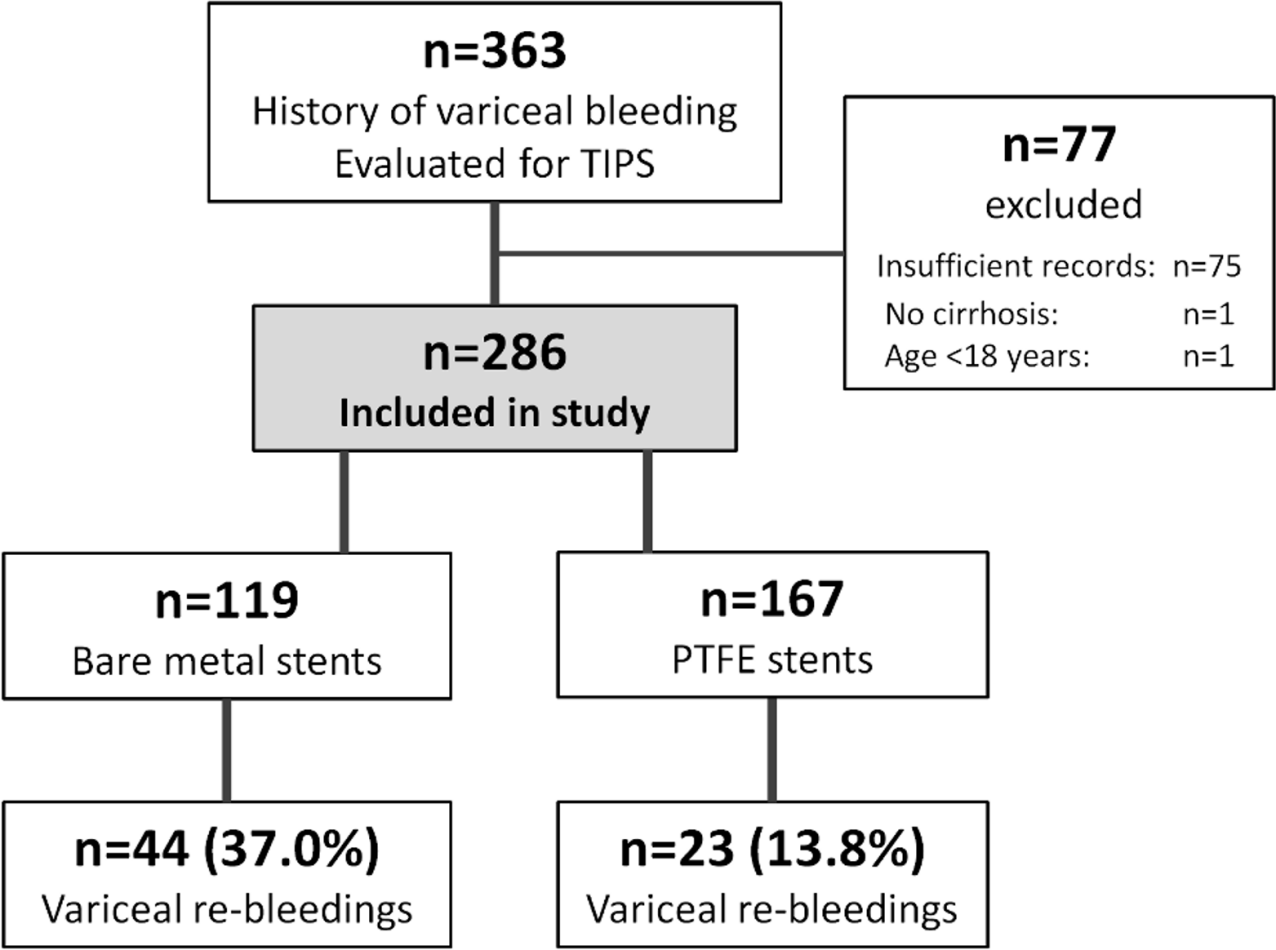
Flowchart of patient inclusion. Abbreviations: TIPS, transjugular intrahepatic portosystemic shunt.

**Table 1:** Patient characteristics.

Patient characteristics	All patients	Bare metal stents	ePTFE stents	p-value
Number of patients	286	119	167	
**Age [years]**	55.1 ± 11.1	55.0 ± 11.3	55.2 ± 11.0	0.853
**Sex [m/f, %m]**	210/76, 73%	87/32, 73%	123/44, 74%	0.918
Etiology of liver disease*				
** - Alcohol**	200 (74%)	89 (79%)	111 (70%)	0.437
** - Viral**	30 (11%)	10 (9%)	20 (13%)	
** - Combined alcohol+viral**	27 (10%)	9 (8%)	18 (11%)	
** - other**	15 (6%)	5 (4%)	10 (6%)	
**Types of varices†**				
** - Esophageal varices (EV)**	208 (77%)	88 (80%)	120 (75%)	0.480
** - Gastric varices (GV)**	8 (3%)	2 (2%)	6 (4%)	
** - EV + GV**	55 (20%)	20 (18%)	35 (22%)	
**Elective TIPS implantation¥**	194 (78.2%)	71 (71.0%)	123 (83.1%)	0.023
**Rescue TIPS implantation¥**	41 (16.5%)	22 (22.0%)	19 (12.8%)	0.057
**NSBB therapy at TIPSŦ**	55 (20%)	13 (11%)	42 (27%)	0.001
**Hepatocellular carcinoma**	18 (6.3%)	8 (6.7%)	10 (6.0%)	0.801
**MELD**	11.8 (9.9–14.4)	12.7 (10.7–15.2)	11.2 (9.6–13.7)	0.001
**Na [mmol/l]**	137.3±4.4	137.4±4.8	137.2±4.2	0.707
**Platelets [G/l]**	106 (76–150)	106 (77–161)	106 (71–149)	0.452
**Bilirubin [mg/dl]**	1.44 (0.96–2.21)	1.55 (0.99–2.33)	1.35 (0.91–2.16)	0.191
**Creatinine [mg/dl]**	0.90 (0.77–1.11)	0.97 (0.85–1.15)	0.87 (0.73–1.06)	<0.001
**INR**	1.33 (1.20–1.50)	1.42 (1.21–1.63)	1.30 (1.20–1.42)	0.004
**HVPG prior to TIPS [mmHg]‡**	20.4±6.2	22.7±6.4	19.6±5.9	0.003
**- PPG change [mmHg]‡**	-12.6±5.3	-12.8±5.6	-12.6±5.2	0.835
**- PPG change [%]‡**	-61.7±16	-55.6±14.7	-63.9±15.9	0.001
**Hospitalization after TIPS [days]**	5 (3–9)	6 (3–10)	5 (4–8)	0.436
**Follow-up [months]**	29.8 (7.4–69.4)	39.4 (7.0–99.3)	26.3 (8.4–162.4)	*0*.*127*
**TFS [months]**	46.0 (12.4–104.4)	43.6 (9.3–109.4)	47.4 (15.3–90.2)	0.968

Laboratory and clinical variables prior to TIPS implantation. Abbreviations: ALD, alcoholic liver disease; EV, esophageal varices; GEV, gastroesophageal varices; IGV, isolated gastric varices; MELD, model for end-stage liver disease; INR, international normalized ratio; HVPG, hepatovenous pressure gradient; NSBB, non-selective betablocker; PPG, portosystemic pressure gradient; TFS, transplant-free survival.

(*) Etiology was identified in n = 272 patients (N = 113 in the bare metal stent group, N = 159 in the ePTFE stent graft group).

(†) Location of varices was reported in n = 271 patients (N = 110 in the bare metal stent group, N = 151 in the ePTFE stent graft group).

(¥) Data on TIPS setting (elective versus rescue) was available in n = 248 patients.

(Ŧ) Data on NSBB comedication was available in N = 273 patients (N = 119 from the bare metal stent group, N = 154 from the ePTFE group).

(‡) HVPG/PPG was documented in N = 185 patients (N = 47 in the bare metal stent group, N = 138 in the ePTFE stent graft group)

Bare metal stents were implanted in 119 patients (41.6%), while 167 patients (58.4%) received ePTFE stents. Bare stents were implanted between years 1994 and 2000. The first ePTFE-covered stent was inserted in 1998. Since September 2000, exclusively ePTFE stents have been used. The majority of patients were men (n = 210, 73%) with a mean age of 55.1 (±11.1) years. Most common etiology of cirrhosis was alcoholic liver disease (ALD) with 74%, followed by chronic viral hepatitis (HBV or HCV) in 11% and mixed alcoholic and viral etiology in 10%. Other etiologies were found in 6% of patients. In n = 14 patients (5%), etiology of cirrhosis could not be determined. The location in endoscopy prior to TIPS was reported in n = 271 (95%) patients. The most frequent location was Most patients had exclusively esophageal varices (EV: n = 208, 77%), while n = patients (20%) showed both esophageal and gastric varices (GEV) and only n = 8 (3%) patients had isolated gastric varices (GV). In 15 patients, the location of varices was not reported. N = 41 (16.5%) patients underwent rescue TIPS implantation, n = 194 (78.2%) patients underwent elective TIPS implantation, while the TIPS setting was unknown in n = 31 patients. Fifty-five patients (20%) patients were on NSBBs prior to TIPS implantation and n = 18 patients (6.3%) had a diagnosis of HCC prior to TIPS implantation.

Median hospitalization time after TIPS implantation was 5 days (IQR: 3–9 days), and median follow-up was 895 days (IQR 223–2082 days). In total, 206 patients (72%) died within the follow-up period and 22 patients (8%) underwent OLT (S1 Table).

### Differences between patients with bare metal and ePTFE-covered stents (Table 1)

Age and sex were comparable between patients with bare metal and ePTFE stent implantation, as was the median hospitalization time after TIPS implantation (6 days, IQR 3–10 days vs. 5 days, IQR 4–8 days; p = 0.436). Similarly, etiology of cirrhosis and location of varices (EV: 74% vs 72%, GV: 2% vs 4%, GV+EV 17% vs 21% in bare metal vs. ePTFE stents, respectively; p = 0.480) did not differ between the groups. More patients received “elective” TIPS in the ePTFE group (83.1% vs. 71.0%; p = 0.023), while bare metal stents tended to be more often used in the “rescue TIPS” setting (22.0% vs. 12.8%; p = 0.057).

While MELD-Na scores were similar between groups, MELD was significantly higher in patients who received bare stents as compared to patients who received ePTFE stents (median 12.7 vs. 11.2 points, p = 0.001), as were its components creatinine (median 0.97 mg/dl vs. 0.87 mg/dl, p<0.001) and INR (1.42 vs. 1.30, p = 0.004), but not bilirubin (median 1.55 vs. 1.35 mg/dl, p = 0.191). Of note, significantly more patients in the ePTFE group than in the bare stent group received NSBB prophylaxis prior to TIPS implantation (ePTFE: 27% vs. bare metal: 11%; p = 0.001).

### Efficacy of TIPS to prevent variceal rebleeding (Table 2, Figs 1 and 2, S1 Table, S1 Fig)

**Fig 2:**
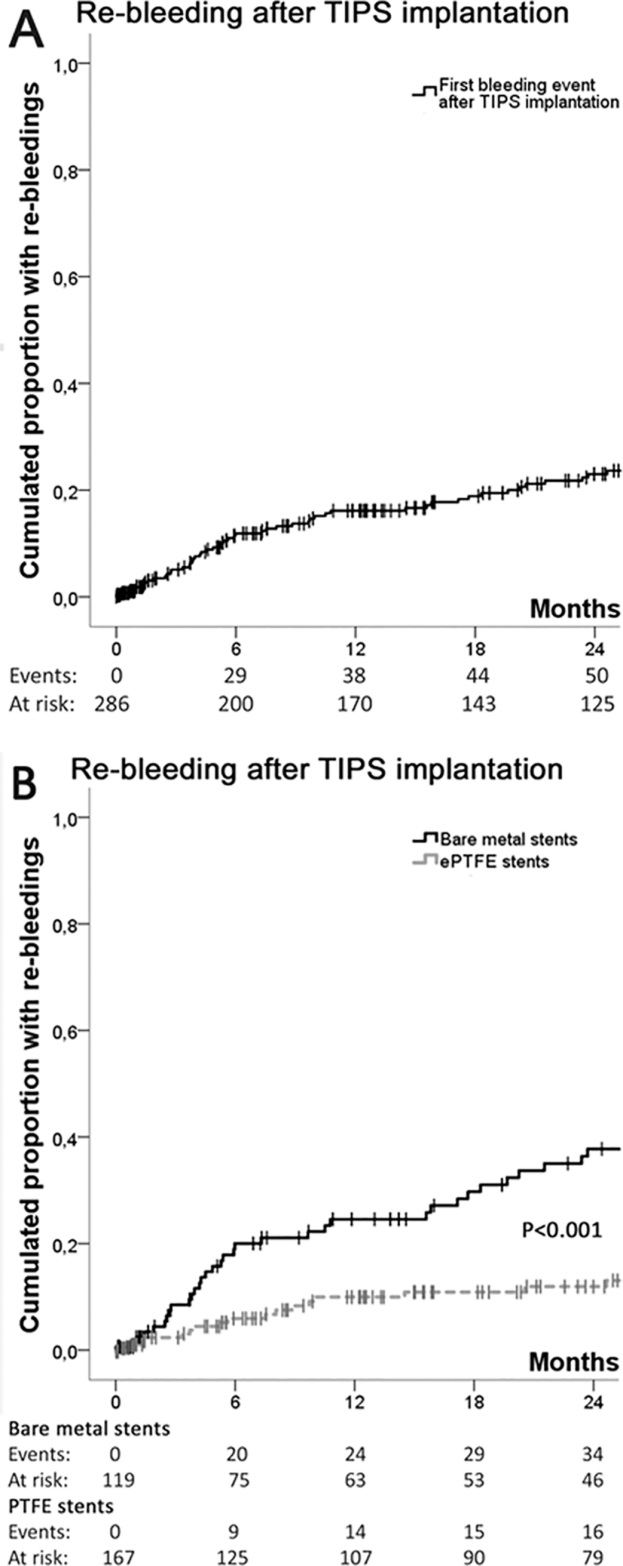
Re-bleeding rates and re-interventions after TIPS implantation. (A). Rebleeding rates after TIPS implantation. (B). Bleeding recurrence after TIPS implantation, by type of implanted stent.

A total of 67 patients (23%) had at least one re-bleeding episode following TIPS-implantation, which occurred significantly less often in patients with ePTFE stents (n = 23, 14%) than in patients with bare stents (n = 44, 37%) (p<0.001 on log-rank analysis). 52 (78%) re-bleedings occurred within the first year after the TIPS implantation. Among patients experienced rebleeding within the first year, two-thirds had a bare metal stent implanted, while only one third had ePTFE stents implanted (p<0.001). Furthermore, in the ePTFE group, the re-bleeding rate reached a plateau at one year after implantation (Fig 2B), while over 20% of patients with bare metal stents re-bled between 12 and 18 months after intervention.

**Table 2:** Risk factors for re-bleeding within the first year after TIPS implantation.

	No re-bleeding within Y1 N = 234	Re-bleeding within Y1 N = 52	p value univariate
Sex [m/f (%male)]	171/63 (73%)	39/13 (75%)	0.776
Age [years]	55 ± 11.2	55 ± 10.6	0.902
Etiology of cirrhosis			0.111
- **Alcohol**	159 (68%)	41 (79%)	
- **Viral**	25 (11%)	5 (10%)	
- **Combined alcohol+viral**	21 (9%)	6 (12%)	
- **Other**	29 (12%)	0 (0%)	
**Types of varices prior to TIPS:**	**Presence of GV: 0.044**		
**Esophageal varices (EV)**	164 (70%)	44 (85%)	Overall: 0.157
**Gastric varices (GV)**	**8 (3%)**	**0 (0%)**	
**EV + GV**	**49 (21%)**	**6 (12%)**	
**unknown**	13 (6%)	2 (4%)	
**Stent type:**			
- **Bare metal (n = 119)**	84 (36%)	35 (67%)	**<0.001**
- **PTFE (n = 167)**	150 (64%)	17 (33%)	
**NSBB post-TIPS**	37 (16%)	3/63 (6%)	*0*.*059*
**Pre-existing HCC**	15 (6%)	3 (6%)	1.000
**MELD**	11.9 (10.2–14.5)	11.9 (9.9–14.3)	0.935
**MELD-Na**	13.8 (11.3–18.1)	14.2 (10.7–17.2)	0.703
**Bilirubin**	1.44 (0.97–2.20)	10.40 (0.89–2.32)	0.883
**Creatinine**	0.90 (0.77–1.13)	0.91 (0.78–1.08)	0.807
**Platelets [G/L]**	103 (75–146)	120 (78–182)	0.170
**Serum sodium**	137 ± 4.5	138 ± 4.1	0.688
**INR**	1.33 (1.21–1.48)	1.32 (1.18–1.52)	0.948
HVPG prior to TIPS [mmHg]*	20.5±6.2	20.0±6.1	0.720
Change in PPG [mmHg]*	-12.6±5.3	-12.8±5.2	0.838
Change in PPG [%]*	-61.4%	-63.7%	0.505
**Multivariate analysis**	**OR**	**95% CI**	**p value**
**ePTFE vs. Bare metal stents**	**0.259**	**0.123–0.542**	**<0.001**
**Presence of GV**	0.427	0.154–1.184	0.102
**MELD (per point)**	0.947	0.868–1.034	0.224
**Female vs. male sex**	1.110	0.483–2.554	0.438
**Age (per year)**	0.996	0.962–1.032	0.836
**NSBB post-TIPS**	0.916	0.243–3.460	0.898

Abbreviations: ALD, alcoholic liver disease; EV, esophageal varices; GEV, gastro-esophageal varices; IGV, isolated gastric varices; ePTFE, expanded tetrafluoroethylene-covered; NSBB, non-selective betablocker; HCC, hepatocellular carcinoma; MELD, model for end-stage liver disease; INR, international normalized ratio; HVPG, hepatovenous pressure gradient; PPG, portosystemic pressure gradient.

(*) HVPG/PPG was documented in N = 185 patients (N = 161 in non-re-bleeding patients, N = 24 in re-bleeders)

In addition to type of TIPS (bare vs ePTFE), also the location of varices (esophageal vs gastric) was a prognostic factor for re-bleeding. EV were more prevalent in patients who re-bled while gastric varices were less prone to rupture after TIPS (24% vs. 12%, p = 0.044). Patients who did not re-bleed within one year after TIPS implantation were more often discharged with an NSBB prescription (in 16%) than patients with re-bleeding (only in 6%). In other words, one-year re-bleeding rate in patients with continued intake of NSBB was 8% (n = 3/40), while it was 20% (n = 49/246) in patients without NSBBs after TIPS (p = 0.059).

In total, 67 patients (23%) needed TIPS revision, 52 (78%) of which required stent extension or dilation and 15 (22%) required a reduction in stent diameter. Stent dilations were performed significantly more often in bare metal stents than in ePTFE stent grafts (91% vs 66%, p = 0.022); conversely, stent reductions were more common in ePTFE stents.

Most stent revisions were required within the first 7 months (Fig 2C). At two years, the proportion of patients with bare metals stents who required stent dilatations was almost 40%, which was only necessary in 20% of ePTFE TIPS (log rank: p = 0.011).

In multivariate analysis, stent type was the only independent risk factor for re-bleeding within 1 year, while other factors (sex, age, MELD, presence of gastric varices, NSBB therapy after TIPS implantation) did not show an independent association with rebleeding. The use of an ePTFE stent reduced the risk of one year bleeding by 74.1% (OR 0.259, 95% CI 0.123–0.542, p<0.001) as compared to bare metal stents.

### Outcome, mortality and transplant-free survival (Fig 3, Table 3, S1 Table, S2 Fig)

During follow-up 22 patients (7.7%) underwent OLT (12% of patients with bare stents and 9% of patients with ePTFE stents), 16 patients (6%) developed HCC (5% with bare metal and 6% of patients with ePTFE stents) and 206 patients (72%) died. Notably, the overall incidence of overt HE was 22% and did not differ between stent types (20% for bare metal and 24% for ePTFE TIPS; p = 0.449).

**Fig 3:**
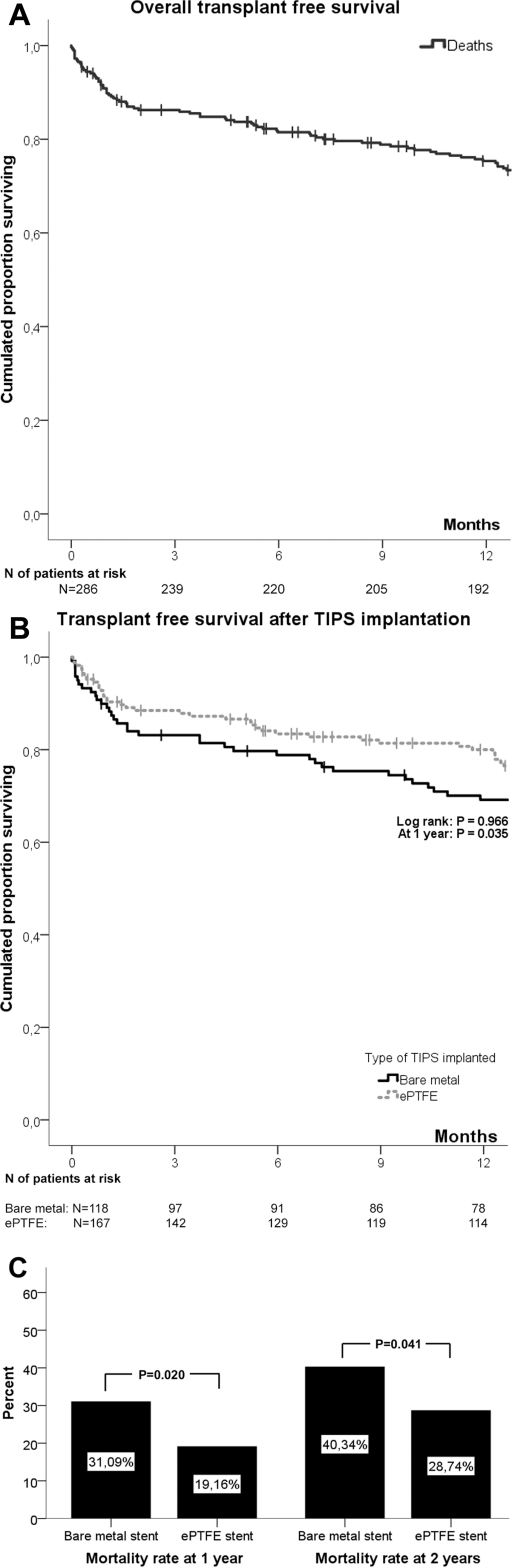
Transplant-free survival and mortality after TIPS implantation. (A). Transplant-free survival. (B). Transplant-free survival, by type of implanted stent. (C). Mortality rates at 1 and 2 years, by type of implanted stent.

**Table 3:** Independent risk factors for mortality.

	OR	95% CI	p value
**MELD (per point)**	**1.060**	**1.009–1.114**	**0.022**
**Age (per year)**	*1*.*019*	*0*.*998–1*.*040*	*0*.*083*
**ePTFE vs bare metal stent**	1.284	0.825–1.998	0.268
**Rescue TIPS setting (<72h)**	0.747	0.391–1.426	0.376
**NSBB treatment (after TIPS)**	1.293	0.709–2.357	0.402
**Female vs. male sex**	1.099	0.697–1.732	0.685
**HVPG (per mmHg)**	0.997	0.960–1.037	0.899
**Re-bleeding**	0.978	0.590–1.622	0.932

Multivariable Cox-regression model including n = 184 patients with a complete data set. Abbreviations: MELD, model for end-stage liver disease; NSBB, non-selective beta-blockers; HVPG, hepato-venous pressure gradient; ePTFE, expanded polytetrafluorethylene-covered, OR, odds ratio; CI, confidence interval.

Bleeding-related mortality at 6 weeks was 12%. A similar proportion of patients with bare metal stent (14%) and with ePTFE TIPS (10%) experienced bleeding-related death (p = 0.313). Mortality at both 1 and 2 years was considerably lower after ePTFE TIPS insertion than after bare metal stent implantation (19% vs 31%, p = 0.020 and 29% vs. 40%, p = 0.041, respectively. However, transplant-free survival did not differ between stent groups (Fig 3B, log-rank p = 0.966).

On multivariate analysis of independent risk factors for mortality, only MELD score (OR 1.060 per point, 95% CI 1.009–1.114, p = 0.022) was an independent risk factor for mortality. While older age tended to be associated with mortality (OR 1.019 per year, 95% CI 0.998–1.040, p = 0.083), gender, stent type and recurrence of variceal bleeding did not have an independent effect on survival, and neither did HVPG or NSBB prescription after TIPS implantation.

We further conducted a competing risk analysis to assess actual differences in clinical outcomes after TIPS–considered variceal rebleeding, OLT, and death as competing risks (S2 Fig). Competing risk analysis showed that ePTFE TIPS significantly reduced the risk of variceal re-bleeding (HR: 0.355, 95% CI: 0.215–0.587, p<0.0001), while the risk for death (p = 0.260) or need for OLT (p = 0.860) did not significantly differ between stent types.

## Discussion

Here we report our two decade experience using TIPS for the prevention of variceal re-bleeding in a large cohort of 286 patients. We confirmed superior patency rates and lower re-bleeding rates and lower mortality with ePTFE-covered stents vs. bare metal stents at one and two years after TIPS Implantation. However, only MELD score and age emerged as predictors for mortality.

While most recent studies have focused either on ‘early TIPS’ placement within <72 hours [22] or on technical issues.[23–25], one recent prospective controlled trial from 2016 randomized n = 72 patients to ePTFE-TIPS vs standard prophylaxis with NSBBs and endoscopic band ligation for secondary prophylaxis of rebleeding.[26] This study demonstrated superior bleeding control with TIPS, but no effect on survival.[26] These results were in line with a recent meta-analysis that included mostly studies from 2000–2010[27], and one recent randomized trial from 2015 that focused on cirrhotic patients with portal vein thrombosis.[28]

Outcomes including re-bleeding rates and survival were primarily assessed at 1 year, since other factors than TIPS type are likely contributing more to the long-term outcome, i.e. recurrent/active drinking or successful antiviral treatment of viral hepatitis: variceal re-bleeding occurred more often in the bare metal stent group than in the ePTFE TIPS group, with 1-year re-bleeding rates of 29% in the bare metal group and 10% in the ePTFE group (p<0.001). Patients treated with ePTFE stents also showed significantly lower in-hospital and 1-year mortality in univariate analysis than patients receiving bare metal stents.

However, the two groups were not randomized and thus, did not show equal patient characteristics. The MELD score was higher in patients with bare metal stents, while more ePTFE patients had been treated with standard of care NSBB plus EBL prior to TIPS implantation. Furthermore, ePTFE stents were exclusively used during the more recent years, and thus, some of the differences between ePTFE and bare metal stents in univariate analysis might also be partially attributable to differences in the management (e.g. medical therapy) of cirrhotic patients. Moreover, in recent years stent diameters have become smaller, as smaller diameters of 8mm appear to have similar clinical effect regarding the prevention of variceal re-bleeding with lower risk of HE.[25,29] All these factors may have contributed to the improved 1- and 2-year mortality respectively transplant-free survival in the more recent cohort of ePTFE TIPS patients. This was also reflected by the multivariable Cox regression model, which revealed MELD as the only independent predictive parameter for survival while the type of TIPS did not attain statistical significance. A survival benefit for TIPS was also not confirmed in the competing risk analysis that still demonstrated a significantly lower risk of re-bleeding with ePTFE TIPS (see S2 Fig).

In line with previous data, we confirmed that bare metal stents had a higher rate of dysfunction than covered stents–which is presumably the cause for the excessive re-bleeding rates in the bare stent group.[30,31] Studies have reported a high rate of hepatic encephalopathy (HE) in ePTFE stents. However, we did not find a difference in incidence of overt HE between covered and uncovered stents in our study population. Stent occlusion is a common complication in TIPS and leads to recurrence of complications of portal hypertension such as variceal bleeding.[32] In the study by Haskal et al. 50% of patients needed bare metal stent revision after one year[33], and Masson et al. reported an even higher need for re-interventions of 76.9% with uncovered stents as compared to only 13.8% in the covered stent group.[34] One meta-analysis assumed a higher rate of hepatic encephalopathy (HE) in ePTFE stents due to higher patency rates.[35] However, we did not observe a difference in incidence of overt HE between covered and uncovered stents in our study population, and neither did previous studies.[30,36]

Interestingly, only 6 patients (12%) with gastroesophageal varices and not a single patient with isolated gastric varices experienced re-bleeding after TIPS implantation. However, in multivariate analysis the presence of isolated or additional gastric varices was not associated with a decreased risk of re-bleeding. The effectivity of TIPS for managing bleeding isolated gastric and gastroesophageal varices has been validated in a meta-analysis, though it has also shown a higher collective morbidity (i.e. hepatic encephalopathy) as compared to variceal sclerotherapy [37]. Our data underline the effectiveness of TIPS in controlling variceal re-bleeding from gastric varices, where endoscopic treatments are often technically challenging.

Given the retrospective nature of this study, our data recording was limited to the available medical records and documentation. For instance, since we wanted to avoid a reporting bias as CPS was not evaluable in earlier patients, we decided to use the MELD score (which is based on laboratory values and not on subjective parameters) to assess severity of liver dysfunction. Despite both electronic and paper databases being thoroughly screened, we cannot exclude some degree of underreporting due to the inherent limitations of non-standardized clinical documentations outside of clinical studies. Thus, we could not adjust for some potential confounding factors such as alcohol consumption, which is difficult to assess even in prospective studies and especially in patients with ALD. Furthermore, medical treatment and patient management have improved over the past few decades in all fields of medicine, including the treatment of portal hypertension. This effect is demonstrated by the higher rate of elective TIPS implantations for secondary prophylaxis in later years (since ePTFE stent grafts have been in use) as compared to earlier years, when rescue TIPS due to uncontrolled bleeding was a common indication. The actual impact of this bias is hard to evaluate, but should certainly be considered when interpreting our study results.

In summary, the results of our study confirm the effectiveness of ePTFE-covered stents for the prevention of variceal re-bleeding as ePTFE-covered TIPS show better shunt patency and less need for re-interventions as compared to bare metal stents. Since the favorable clinical course with ePTFE-TIPS as compared to bare metal stents seem to translate into improved survival, TIPS implantation for prevention of variceal bleeding should be exclusively performed using covered stent grafts.

## Supporting information

S1 Table Outcome after TIPS-implantation. Abbreviations: ePTFE, expandable polytetrafluoroethylene covered stent graft; HE, hepatic encephalopathy; HCC, hepatocellular carcinoma; OLT, orthotopic liver transplantation. (*) Data available in N = 280 patients.
(DOCX)

S1 Fig Need for re-intervention after TIPS-implantation, by type of implanted stent. Abbreviations: ePTFE, expandable polytetrafluoroethylene covered stent graft.
(TIF)

S2 Fig Competing risk analysis with re-bleeding, liver transplantation, and death as competing risks. (A) Competing risk analysis for all included patients. (B) Competing risk analysis comparing risks in patients receiving ePTFE vs bare metal stents. Abbreviations: ePTFE, expandable polytetrafluoroethylene covered stent graft; BARE, uncovered/bare metal stents; OLT, orthotopic liver transplantation.
(PNG)

## Abbreviations

AIH: autoimmune hepatitis
ALD: alcoholic liver disease
ALT: alanine-aminotransferase
AST: aspartate-aminotransferase
CRP: C-reactive protein
(e)PTFE: (expandable) polytetrafluoroethylene
EV: esophageal varices
Hb: hemoglobin
HVPG: hepatic venous pressure gradient
IGV: isolated gastric varices
INR: international normalized ratio
MELD: model for end-stage liver disease
HE: hepatic encephalopathy
OLT: orthotopic liver transplantation
PHT: portal hypertension
PPG: portosystemic pressure gradient
TFS: transplant free survival
TIPS: transjugular intrahepatic portosystemic shunt
WBC: white blood cell count
