# Supplementary material for: Transjugular intrahepatic portosystemic shunts (TIPS) for the prevention of variceal re-bleeding – A two decades experience
Source: PLoS One. 2018 Jan 9;13(1):e0189414. doi: 10.1371/journal.pone.0189414 (PMC5760018; doi:10.1371/journal.pone.0189414)
Supplement: S1 Table — Abbreviations: ePTFE, expandable polytetrafluoroethylene covered stent graft; HE, hepatic encephalopathy; HCC, hepatocellular carcinoma; OLT, orthotopic liver transplantation. (*) Data available in N = 280 patients. (DOCX) [file pone.0189414.s001.docx]

## TABLES

**Supplementary Table-S1. Outcome after TIPS-implantation.**

|  | Overall  n=286 | Bare metal  n=119 | ePTFE  n=167 | p value |
| --- | --- | --- | --- | --- |
| Patients with TIPS revisions   - extension/dilatation - diameter reduction | 67 (23%)  52 (78%)  15 (22%) | 32 (37%)  29 (91%)  3 (9%) | 35 (21%)  23 (66%)  12 (34%) | **0.024**  **0.022**  *0.081* |
| Patients with re-bleedings | 67 (23%) | 44 (37%) | 23 (14%) | **<0.001** |
| Overt HE | 64 (22%) | 24 (20%) | 40 (24%) | 0.449 |
| HCC | 16 (6%) | 6 (5%) | 10 (6%) | 0.731 |
| OLT | 22 (7.7%) | 11 (9.3%) | 11 (6.6%) | 0.554 |
| Deaths   - In-hospital* - Within 6 weeks - Within 1 year - Within 2 years | 17 (6%)  34 (12%)  69 (24%)  96 (34%) | 13 (11%)  17 (14%)  37 (31%)  48 (40%) | 4 (2%)  17 (10%)  32 (19%)  48 (29%) | **0.004**  0.313  **0.020**  **0.041** |

**´**

Abbreviations: ePTFE, expandable polytetrafluoroethylene covered stent graft; HE, hepatic encephalopathy; HCC, hepatocellular carcinoma; OLT, orthotopic liver transplantation.

(*) Data available in N=280 patients.
