# Supplementary figures and images for: Transjugular intrahepatic portosystemic shunts (TIPS) for the prevention of variceal re-bleeding – A two decades experience
Source: PLoS One. 2018 Jan 9;13(1):e0189414. doi: 10.1371/journal.pone.0189414 (PMC5760018; doi:10.1371/journal.pone.0189414)

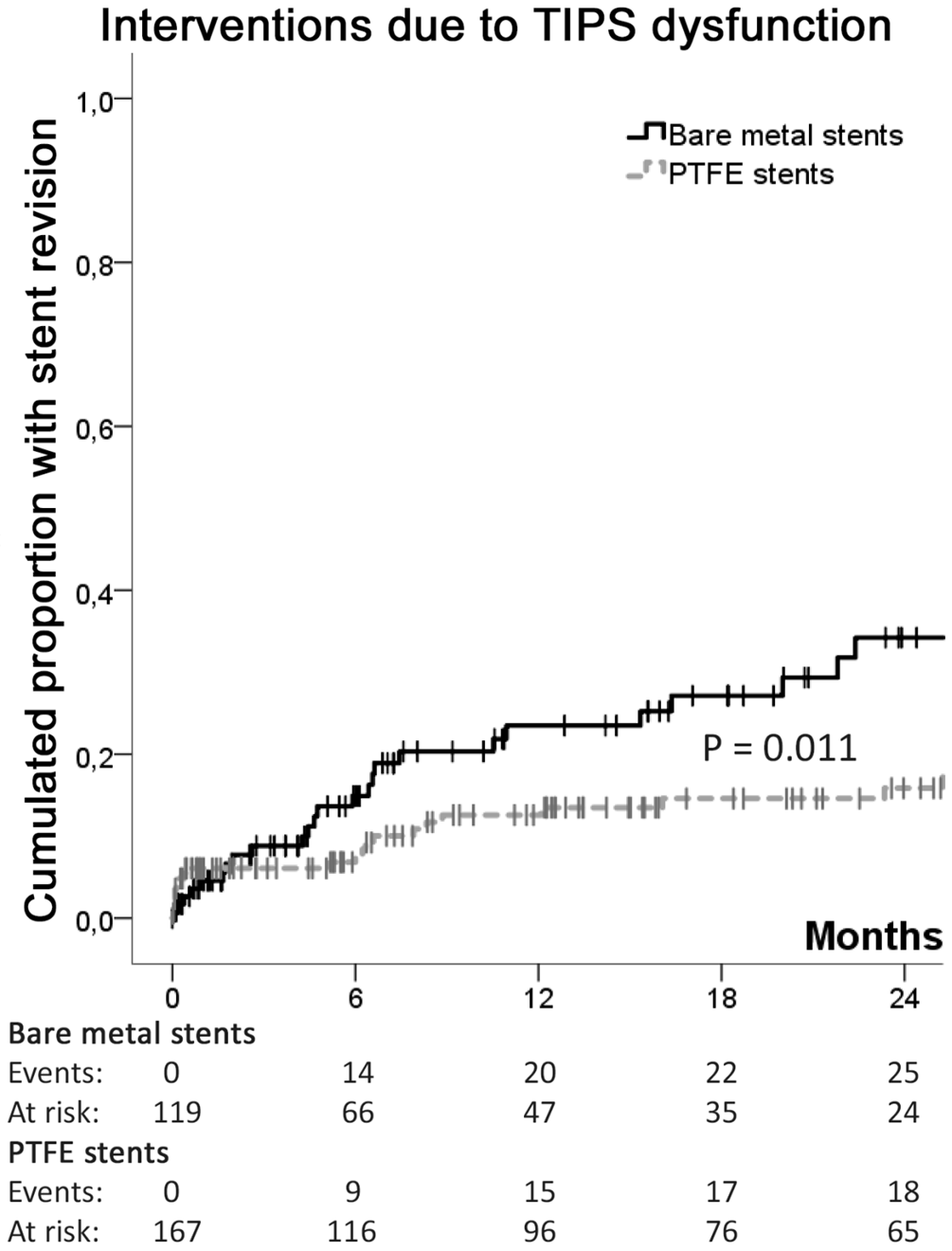

Supplement: S1 Fig — Abbreviations: ePTFE, expandable polytetrafluoroethylene covered stent graft. (TIF) [file pone.0189414.s002.tif]

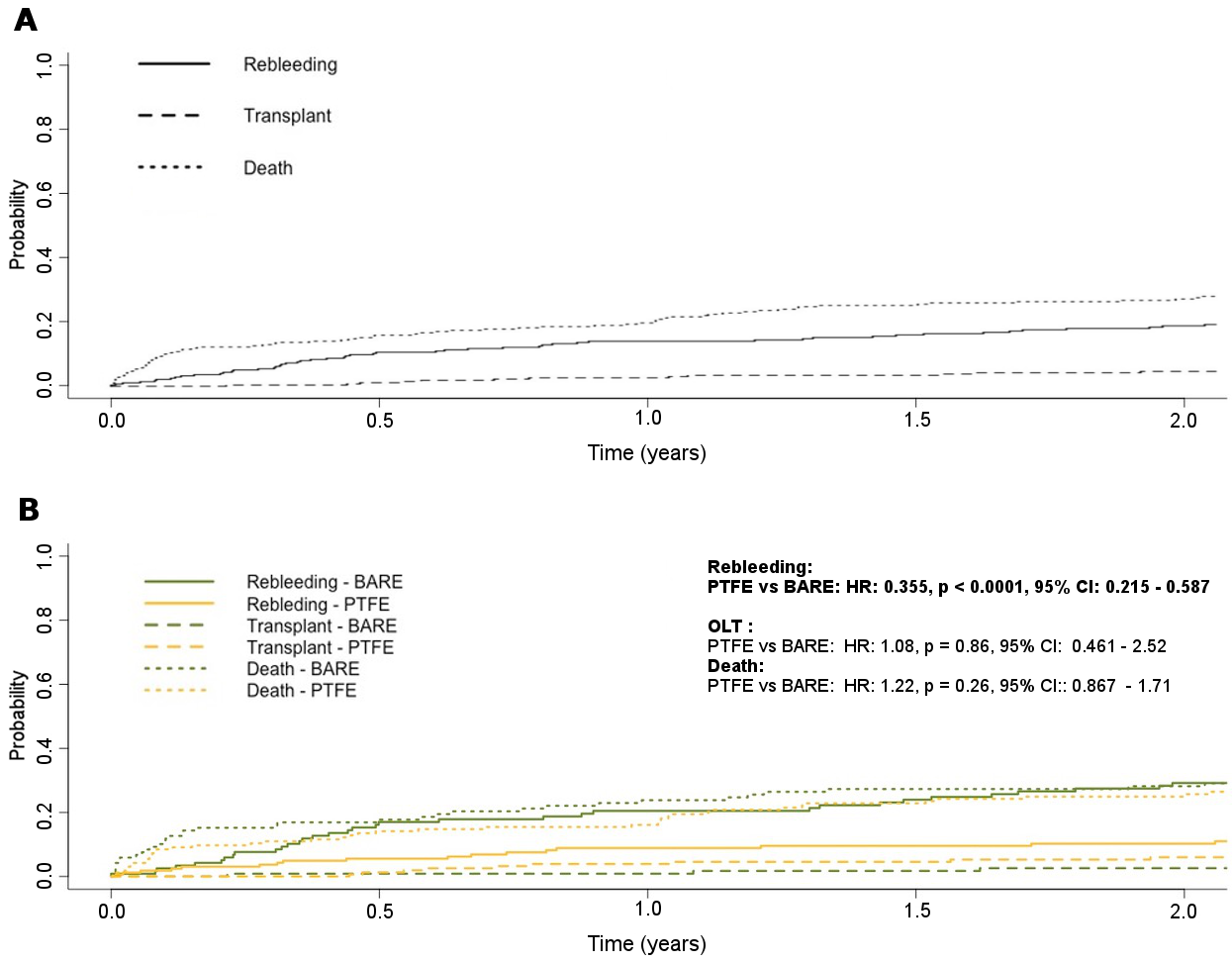

Supplement: S2 Fig — (A) Competing risk analysis for all included patients. (B) Competing risk analysis comparing risks in patients receiving ePTFE vs bare metal stents. Abbreviations: ePTFE, expandable polytetrafluoroethylene covered stent graft; BARE, uncovered/bare metal stents; OLT, orthotopic liver transplantation. (PNG) [file pone.0189414.s003.png]
